# Supplementary material for: Identification of skill in an online game: The case of Fantasy Premier League
Source: PLoS One. 2021 Mar 3;16(3):e0246698. doi: 10.1371/journal.pone.0246698 (PMC7928501; doi:10.1371/journal.pone.0246698)
Supplement: S1 File — (PDF) [file pone.0246698.s001.pdf]

Identification of skill in an online game: The case of Fantasy Premier League

Joseph D. O'Brien, James P. Gleeson, and David J. P. O'Sullivan.

S1 Note. Summary of Rules of Fantasy Premier League

The decisions made by the managers of Fantasy Premier League are governed by a stringent set of rules [31]. The initial restrictions of the game is that the manager must select a squad of 15 players consisting of two goalkeepers, five defenders, five midfielders, and three forwards. The total value of these players may not exceed £100M and a further restriction is that no more than three players from one club may appear in a given squad. Each week the manager must then select a starting 11 players which must include one goalkeeper and a minimum of three defenders, three midfielders, and one forward, this restriction is known as the formation criterion. These players are the ones whose performance contributes to the managers' points total. The remaining players feature on the 'bench' and are ordered by the manager such that if one of their starting players does not appear the first placed bench players' points are given to the manager (provided the formation criterion remains satisfied by said first choice bench player).

Players selected by a manager are rewarded points based upon their statistical performance during the physical matches they compete in. Points for specific actions vary by the players' position, for example, a defender receives more points for scoring a goal than a forward due to the relative rarity of such an event. In S1 we show the points per position for each of the possible actions the players may be rewarded/penalised for. For a goalkeeper or defender to be classified as keeping a clean sheet they must have played at least 60 minutes, excluding stoppage time. For example, if a defender was substituted in the physical game with the score at 0-0 in the 63rd minute, and their team proceeded to concede a goal then the defender in question would receive clean sheet points but the remaining players would not. Also, in the case of a goal scored directly from a set piece i.e., a free-kick or penalty, the player who was fouled in the awarding of the set piece receives the assist.

Managers may proceed to make changes to their team between gameweeks which involves *transferring* a player from their team for another with the same position. Each week a manager is entitled to one such transfer known as a *free transfer*. Additional transfers may be made but at a cost of four points each from their points total. If the manager does not make use of their free transfer the following gameweek they may then make two free transfers however it is not possible to accumulate more than two free transfers. The aforementioned restrictions regarding positions, clubs, and value must be satisfied for each transfer.

In terms of the points amassed by the managers observed in the study we show the points totals and the number of manager who obtained them in panel (a) of S1. We firstly comment on the skewness of the distribution with the number of managers obtaining a certain number of points decreasing quickly as the points becomes larger. The large gap between the points obtained by the overall winner (2659) and those of second place (2602) is also interesting. The distribution of points within each tier is shown in S1(b), we see the same skewness being present in each of these distributions, and also the presence of outliers among the top ranked positions. Summary statistics for each tier and also all managers are given in S2 and S3. To view how the ranks of managers change over the course of the season we show the flow of manager position in S2. All managers are considered in panel (a) while those who finished in the top 10^4 positions are shown in (b). We see the competition for the top positions by the fact that no manager who is

S1 Table: Points awarded to players based on action by position.

Action	Goalkeeper	Defender	Midfielder	Forward
For playing up to 60 minutes	1	1	1	1
For playing 60 minutes or more	2	2	2	2
For each goal scored	6	6	5	4
For each assist	3	3	3	3
For keeping a clean sheet	4	4	1	-
For every 3 saves made	1	-	-	-
For each penalty saved	5	-	-	-
For each penalty missed	-2	-2	-2	-2
For every two goals conceded	-1	-1	-	-
For each yellow card	-1	-1	-1	-1
For each red card	-3	-3	-3	-3
For each own goal	-2	-2	-2	-2

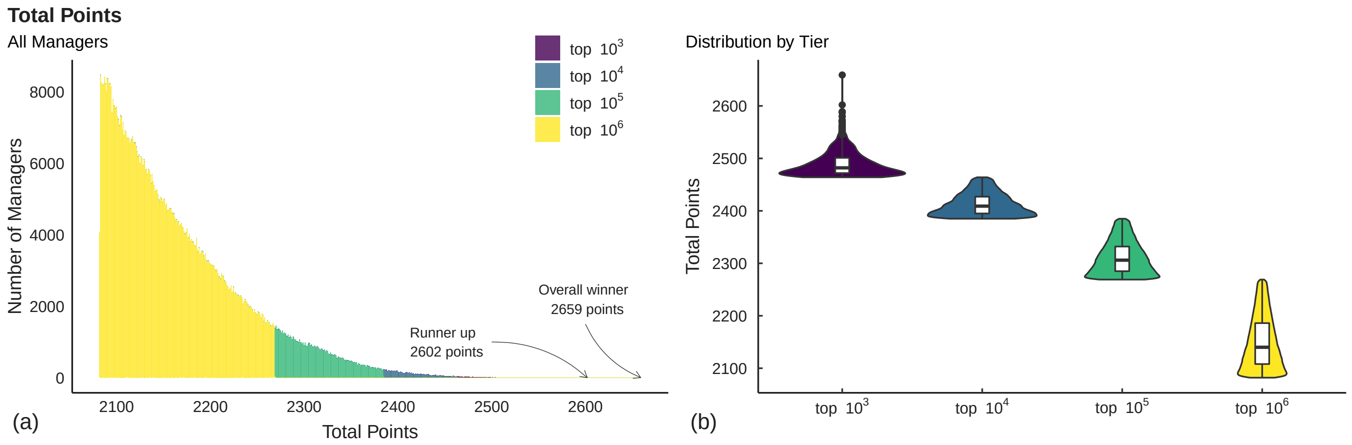

S1 Fig. Summary of points obtained by managers in the 2018/19 season. (a) The number of managers that obtained each points total where the bins are by tier. The overall winner (2659 points) and second place manager (2602) are highlighted. (b) Distribution of points totals earned by each tier.

outside the top 10^6 ranks at gameweek 20 finishes within the top 10^6 tier.

S2 Note. Historical Correlations

To obtain the historical data we considered managers taking part in the 2019/20 season of the game for which we found $\approx$ 6 million managers, of whom $\approx$ 3.8 million had taken part in a previous season. We then determined their historical performance in terms of both points earned and overall rank for each season in which they partook in the game. These quantities are the only two available at the historical level, unlike the gameweek level of resolution studied in the main text. We then proceeded to identify the number of managers for whom we have data in each pairwise combination of seasons, shown in the lower elements of S4, and calculated Pearson correlations between their points totals in each case represented by the upper elements of the same table. These correlations are visualised in Fig. 1 of the main text. The two single blocks in the figure represent the winner (who had played in six previous seasons) and the runner-up (three previous seasons).

We also consider the relationship between the number of previous seasons that the managers took part in and their points totals in the 2018/19 seasons as shown in S3. We comment on the small number of managers present in the bottom right corner of the plot, suggesting that managers who have played for multiple years are less likely to have

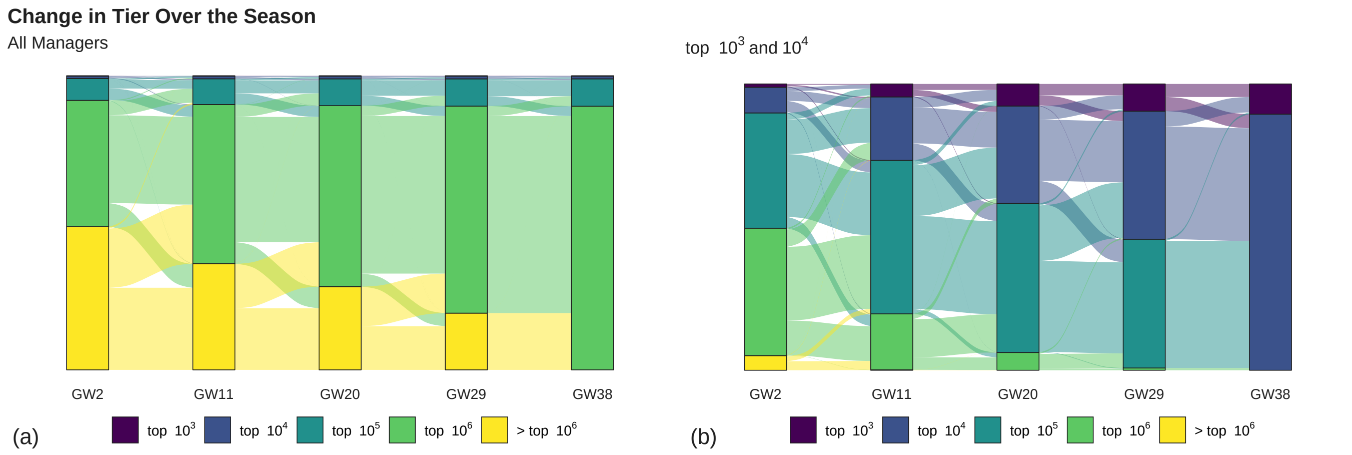

S2 Fig. Alluvial graph describing the flow of manager rank at multiple time points during the season. (a) Change in tier over all managers in the dataset. (b) The same analysis but on those who finished in the top 10^3 and 10^4 tiers.

S2 Table: Average points and standard deviation of points earned for each tier in each gameweek.

	10^3		10^4		10^5		10^6	
GW	Mean	SD	Mean	SD	Mean	SD	Mean	SD
1	88.16	11.73	84.97	12.65	76.84	14.62	63.17	15.06
2	85.44	11.09	83.58	12.18	78.15	14.57	68.49	16.28
3	51.12	8.52	50.04	8.66	49.42	9.73	49.81	11.12
4	51.85	7.74	50.80	7.99	49.70	8.97	47.00	10.23
5	71.64	14.77	68.42	14.67	64.03	14.99	54.69	15.51
6	62.97	8.18	61.84	8.27	59.51	8.89	55.40	9.52
7	62.80	10.36	60.83	10.22	58.13	10.61	53.96	11.33
8	70.82	13.01	66.73	13.06	62.52	13.41	56.79	13.86
9	45.70	7.82	44.79	8.39	43.35	9.12	42.78	10.28
10	75.62	11.34	73.70	12.02	70.56	13.93	65.21	15.70
11	70.94	12.03	68.71	12.62	65.65	13.31	59.63	14.15
12	62.71	7.19	60.57	7.87	57.62	8.58	53.44	8.97
13	48.36	10.92	48.18	11.27	49.19	11.56	50.79	11.99
14	59.28	7.91	58.17	8.35	56.20	9.07	53.60	10.26
15	60.74	11.77	58.48	11.72	55.27	11.95	51.20	12.16
16	72.55	16.74	66.80	16.36	62.70	16.04	58.82	16.41
17	55.06	9.12	53.83	9.73	51.50	10.43	47.06	11.19
18	59.70	13.33	58.41	13.37	57.85	14.13	57.44	15.02
19	78.08	12.44	75.98	12.60	73.45	12.63	68.13	12.63
20	61.98	11.63	58.77	11.75	55.47	12.43	52.15	12.95
21	56.22	11.29	56.24	11.29	56.49	11.41	56.17	11.61
22	67.43	9.04	65.45	9.47	61.50	9.95	55.73	10.31
23	68.75	8.69	67.69	9.50	65.51	10.69	60.86	12.14
24	47.98	9.15	46.86	9.42	46.36	10.19	45.84	11.15
25	86.11	17.93	81.53	18.19	78.68	18.62	73.11	18.48
26	74.72	10.57	72.30	11.05	69.19	11.97	64.48	13.46
27	42.87	9.86	41.67	9.63	40.97	9.97	39.32	10.57
28	59.38	10.03	58.44	10.65	57.36	11.60	56.91	12.90
29	47.24	8.15	45.75	8.51	45.15	9.10	43.86	9.49
30	63.76	16.04	60.68	16.08	57.02	15.66	53.62	14.90
31	35.11	10.41	35.26	10.96	36.44	11.76	34.57	13.45
32	98.99	12.11	96.09	12.93	90.78	14.46	80.69	15.62
33	71.58	12.06	68.07	13.36	60.56	16.10	46.85	17.59
34	50.87	11.80	51.96	12.95	54.25	14.33	58.19	15.21
35	106.96	17.31	100.75	18.72	89.80	19.20	76.07	15.46
36	100.31	16.82	96.12	16.73	90.28	17.11	82.24	17.22
37	49.19	10.50	48.02	11.16	48.67	12.35	50.77	13.20
38	66.81	11.53	66.20	11.96	64.68	12.63	61.25	13.74

obtained a lower points total, in line with the correlation results obtained in S4. This positive relationship is also evident when one fits a linear regression to the data which suggests each additional year's experience is worth, on average, an additional 22 points.

S3 Note. Financial Analysis

Here we briefly consider implications of the financial aspects of the game. Firstly, the price of players themselves demonstrate some interesting dynamics. We remind the reader that initially the price of each player is set by the developers of the game and this price subsequently fluctuates over the season depending on the supply and demand of managers transferring the player in and out of their teams. A summary of the distribution of the average value over the season for each of the ≈600 players coloured by their corresponding position is provided in panel (a) of S4. We comment on the skewed distribution of points obtained in all cases but in particular for midfielders and forwards. The corresponding points earned over the season by each of the players versus their average price is shown in panel (b). We see that there is, in general, a positive relationship between the price of a player and their corresponding points totals. As per their prices, we see a handful of midfielders and forwards who earn the most points.

The make-up of the managers' squad consists of two goalkeepers, five defenders, five midfielders, and three forwards.

S3 Table: Summary statistics of the points obtained by the managers in the dataset. Both over all managers and the tiers used in this study.

	Tier				
	Everyone	10^3	10^4	10^5	10^6
n	901912	1000	8493	83897	808522
Max	2659	2659	2464	2385	2269
Min	2082	2464	2385	2269	2082
Mean	2167.91	2489.82	2412.68	2310.82	2150.11
Median	2150	2482	2409	2306	2140
Std. dev	71.72	24.21	20.68	30.44	49.42
IQR	96	29	32	47	78

S4 Table: Correlation between a managers' historical performance. The lower triangular elements of the table represent the number of managers who were present in both of the seasons, such that the diagonal elements describe the number of managers for each season for whom we could obtain data. Upper elements of the table represent the pairwise Pearson correlation coefficient between the points obtained by the manager in the two seasons.

**Season**	2018-19	2017-18	2016-17	2015-16	2014-15	2013-14	2012-13	2011-12	2010-11	2009-10	2008-09	2007-08	2006-07
2018-19	3,810,484	0.42	0.36	0.30	0.30	0.26	0.25	0.21	0.20	0.20	0.18	0.13	0.14
2017-18	2,577,419	2,951,332	0.43	0.36	0.34	0.31	0.29	0.25	0.24	0.23	0.21	0.15	0.15
2016-17	1,854,775	1,876,837	2,114,618	0.43	0.41	0.33	0.31	0.28	0.25	0.25	0.21	0.15	0.15
2015-16	1,407,603	1,410,074	1,381,427	1,549,899	0.46	0.36	0.31	0.30	0.27	0.24	0.20	0.15	0.15
2014-15	1,163,917	1,171,594	1,134,400	1,153,343	1,279,695	0.47	0.40	0.36	0.32	0.31	0.25	0.18	0.18
2013-14	963,170	969,670	933,216	939,339	946,672	1,058,033	0.45	0.35	0.30	0.28	0.25	0.19	0.19
2012-13	754,449	758,702	731,609	734,445	733,617	741,797	823,537	0.46	0.37	0.35	0.29	0.23	0.22
2011-12	635,833	638,131	612,120	611,371	606,032	604,716	604,303	692,553	0.45	0.40	0.29	0.25	0.23
2010-11	420,544	421,508	409,055	411,163	409,505	409,952	412,101	440,594	453,150	0.44	0.34	0.27	0.26
2009-10	315,106	315,569	306,943	308,726	307,495	308,039	309,390	329,926	313,801	338,437	0.44	0.34	0.33
2008-09	223,217	223,374	217,829	219,255	218,378	218,595	219,578	233,607	221,887	223,383	239,153	0.37	0.35
2007-08	153,640	153,615	149,788	150,831	150,241	150,298	150,976	160,669	151,297	150,545	150,766	164,385	0.38
2006-07	89,260	89,182	87,197	87,819	87,455	87,504	87,994	93,241	88,130	87,250	86,672	87,762	95,231

S5 demonstrates the proportion of budget spend in these three positions (we have grouped goalkeepers and defenders together) by all managers at GW 1. We observe some remarkable variation in where the budget is spent, with some managers spending over half of their budget on midfield players despite of them only accounting for a third of their squad. Due to the price of players fluctuating throughout the season, like an investor holding a varying stock, the managers' overall team value changes. S6 shows the average team value of the managers by tier over each gameweek of the season along with corresponding 95% intervals of the distribution. As per the main text we fit a linear regression to the total points obtained by all managers as a function of their team value each gameweek, with results shown in S5.

S4 Note. Team Similarity and Cluster Analysis

In this section we show more information regarding the hierarchical clustering analysis described in the main text. S7 shows the scaled within sum of squared errors (WSS) for each number of clusters where the error is calculated using k-means. We decide upon four clusters as the decrease in errors slows down at this point. In order to give equal weighting to each gameweek we firstly calculate the WSS for each gameweek and rescale these before averaging over these rescaled values over all gameweeks.

The sizes of these first three clusters for each tier are shown in S8 and follow a similar pattern to that found for all managers in the main text. The top two tiers are shown in panel (a) and (b), however, do appear to make use of fewer players which may be a function of the smaller number of teams to analyse It may also be further evidence of the higher similarity between the teams in these tiers as suggested in S9, which shows the Jaccard similarity (calculated as in the main text) for each of the tiers versus all other tiers. Finally, for the interested reader we provide the identity of those players who appear in the first cluster when the analysis is performed on all managers in S6. We see frequent appearance of some higher priced players such as Mohammed Salah and Sergio Agüero throughout the season. However, also interesting is the presence of some extremely inexpensive players, in particular Aaron Wan-Bissaka who

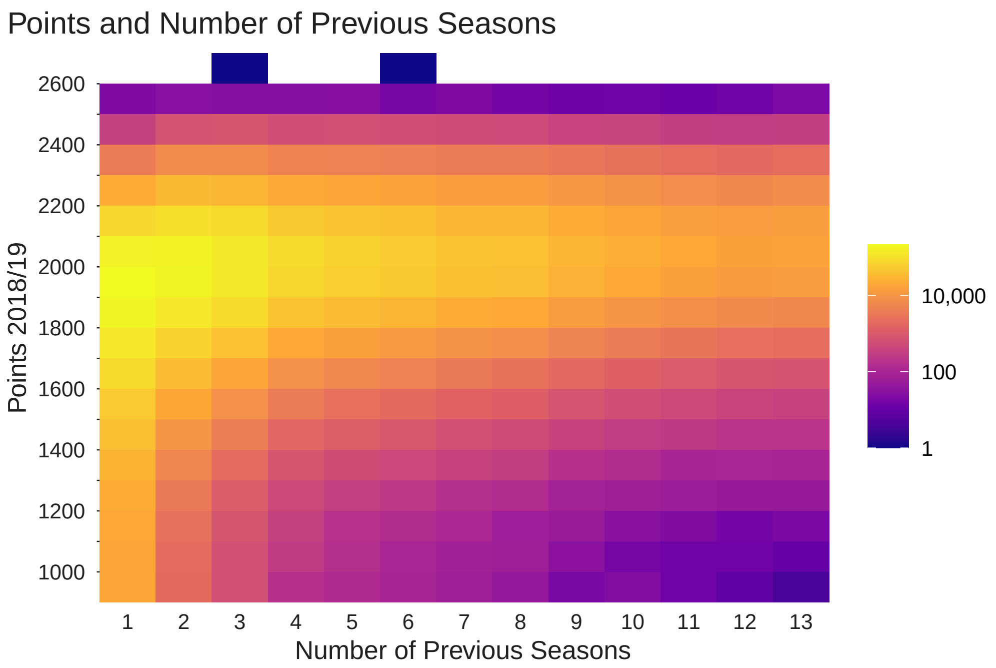

S3 Fig. Manager points in the 2018/19 season versus the number of previous seasons which they had registered for. The bins, each of which cover a 100 point range, are coloured by the number of managers in each, note the logarithmic scale. We comment on the small number present in the bottom right corner (in comparison to the top right corner), which indicates that players who have played for multiple years did not tend to perform poorly.

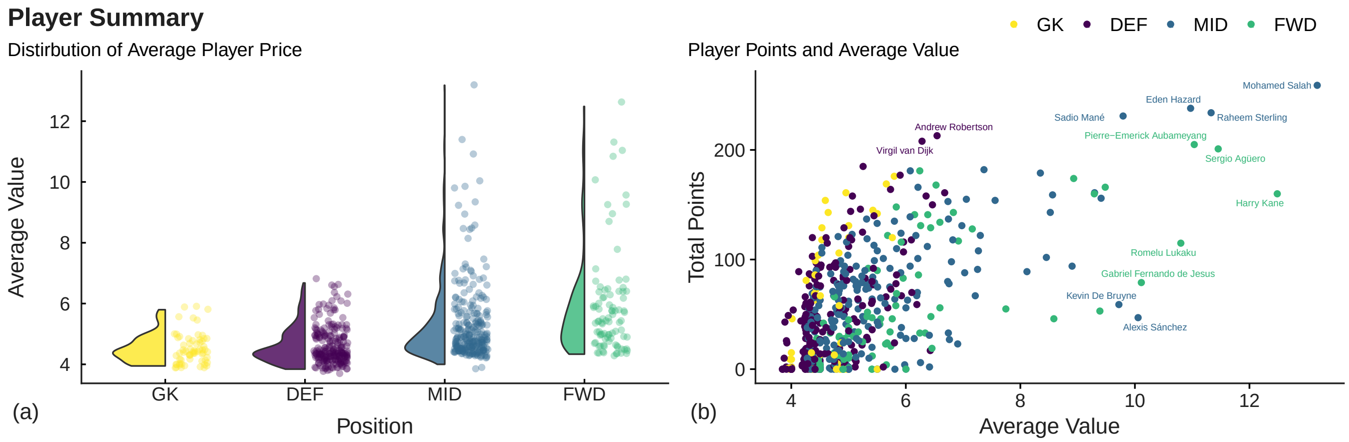

S4 Fig. The value of player values and the corresponding points earned. (a) Distribution of average player price over the course of the season factored by the player position, we comment on the skewed nature of the distribution particularly for midfielders and forwards. (b) The same average value is shown versus the corresponding points earned over the season by said players, which shows the largest points totals being provided by generally the higher priced players. The identities of some players with higher prices and points totals are also shown.

was appearing in his debut campaign and was priced at the cheapest level as a £4M defender but surprisingly made consistent appearances throughout the season, which made him a very attractive option for skilful managers in order to spend more of their budget elsewhere.

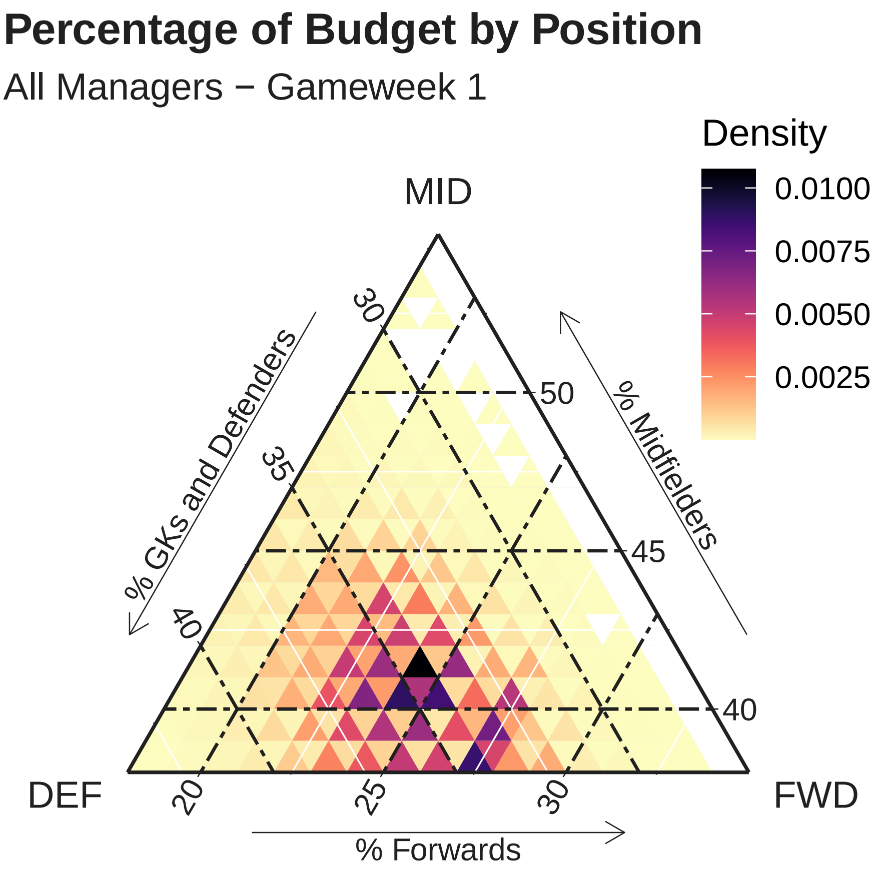

S5 Fig. Ternary diagram demonstrating the make-up of managers' squads in gameweek one. The combination of proportions spent in each position (where DEF represents both goalkeepers and defenders) is shown, where the colour in each bin represents the fraction of managers who used a given combination of proportions.

S5 Note. Chip Usage

As described in the main text we described three chips which essentially are tricks a manager can make use of in any given gameweek (note that more than one chip can not be used in any single gameweek). The chip properties are summarised below

1. **Bench Boost (BB)** - The manager receives the points awarded by all 15 players in their squad in comparison to the usual starting 11 players.
2. **Free Hit (FH)** - The manager may make unlimited changes to their team for one gameweek, at the end of which their team reverts to the squad from the previous GW, under the standard restriction, i.e., they must remain under their budget, satisfy the formation criterion, and have no more than three players from any one club.
3. **Triple Captain (TC)** - For the GW this chip is played in, the captain's points are tripled rather than doubled. If the captain does not play the triple points are awarded to the vice-captain and, as usual, if neither play no one is awarded triple points.

The points obtained for each of these chips, the distributions of which are shown in S10, are calculated by

1. **Bench Boost (BB)** - We identify the four players on the manager bench the week the chip was played and tally their points total.
2. **Free Hit (FH)** - The amount of points the manger received that week is used in this case as the free hit essentially acts like a free week to choose the eleven players of their desire with the aim of maximizing points for one week i.e., no long-term planning is needed.

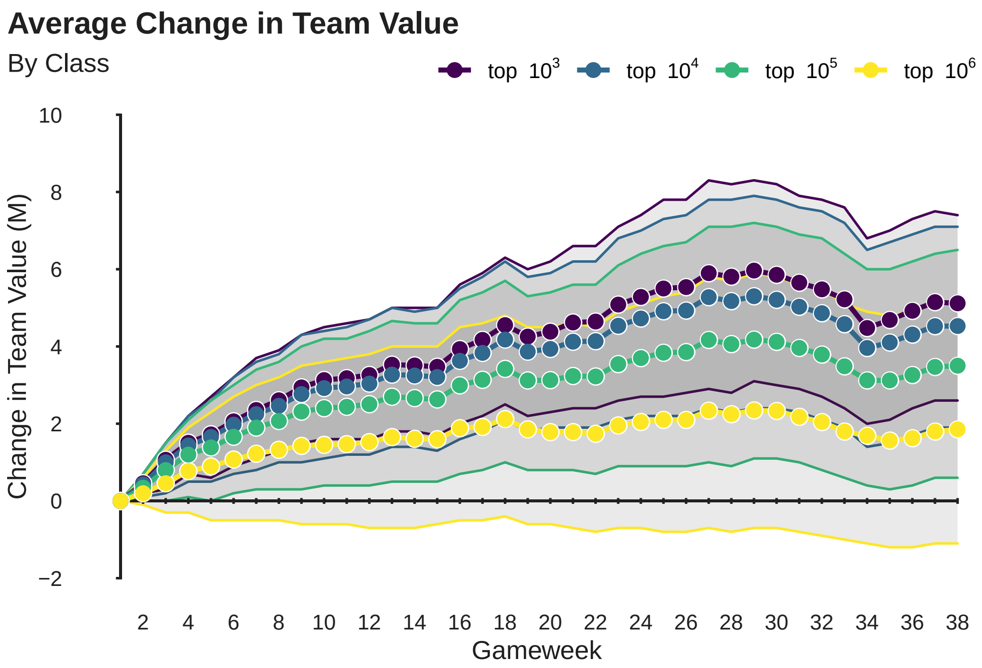

S6 Fig. Average team value along with 95 percentiles for each tier over the season. We comment on the general upward trend, but observe the higher-placed managers having larger team values throughout the season.

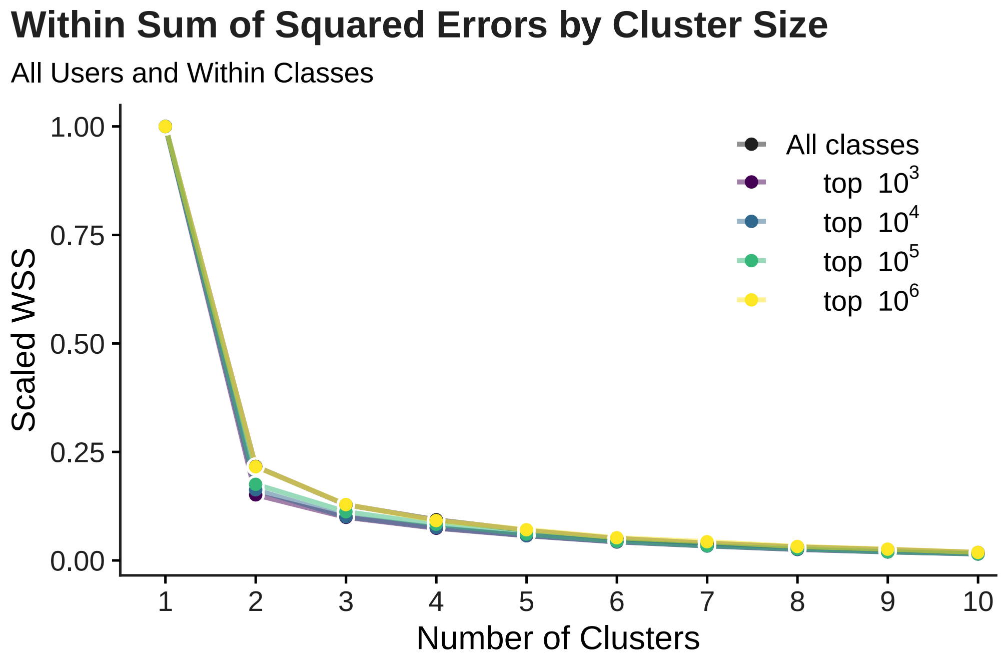

S7 Fig. Scaled within sum of squared errors for the k-means cluster analysis The horizontal axis represents the number of clusters used and the vertical the within sum of squared errors. The measure is calculated for each tier in each of the 38 gameweeks before being rescaled in order to give equal weighting to each gameweek. We note that each tier follows a similar pattern.

3. **Triple Captain (TC)** - The captain's points total is shown in the distribution. We assume they would have chosen this player to be captain regardless of the chip so would have received double points regardless and as such the difference is only the single points score.

S5 Table: Regression coefficients for final points as a function of each additional million pounds in team value at each gameweek over all managers.

GW	Intercept	Co-efficient	p	R^2
1	2167.907	—	—	$< 10^{-6}$
2	2143.709	110.991	$< 10^{-6}$	0.092
3	2141.321	53.410	$< 10^{-6}$	0.107
4	2136.398	38.339	$< 10^{-6}$	0.097
5	2141.617	27.676	$< 10^{-6}$	0.082
6	2138.070	26.221	$< 10^{-6}$	0.094
7	2135.709	24.810	$< 10^{-6}$	0.102
8	2133.034	24.766	$< 10^{-6}$	0.112
9	2133.060	22.865	$< 10^{-6}$	0.124
10	2131.917	23.098	$< 10^{-6}$	0.133
11	2131.612	23.023	$< 10^{-6}$	0.136
12	2132.525	21.688	$< 10^{-6}$	0.130
13	2130.013	21.430	$< 10^{-6}$	0.137
14	2129.650	22.168	$< 10^{-6}$	0.143
15	2130.758	21.695	$< 10^{-6}$	0.138
16	2127.116	20.324	$< 10^{-6}$	0.139
17	2124.623	21.092	$< 10^{-6}$	0.158
18	2120.340	21.016	$< 10^{-6}$	0.167
19	2124.464	21.789	$< 10^{-6}$	0.169
20	2124.513	22.473	$< 10^{-6}$	0.185
21	2124.235	22.474	$< 10^{-6}$	0.198
22	2125.238	22.415	$< 10^{-6}$	0.202
23	2122.807	21.153	$< 10^{-6}$	0.203
24	2122.490	20.379	$< 10^{-6}$	0.204
25	2122.837	19.661	$< 10^{-6}$	0.207
26	2124.070	19.118	$< 10^{-6}$	0.204
27	2122.017	18.063	$< 10^{-6}$	0.202
28	2124.113	17.892	$< 10^{-6}$	0.198
29	2122.244	17.922	$< 10^{-6}$	0.201
30	2122.089	18.120	$< 10^{-6}$	0.198
31	2123.304	18.771	$< 10^{-6}$	0.204
32	2126.808	18.373	$< 10^{-6}$	0.196
33	2130.075	19.057	$< 10^{-6}$	0.195
34	2134.159	18.271	$< 10^{-6}$	0.162
35	2135.771	18.504	$< 10^{-6}$	0.176
36	2133.740	18.915	$< 10^{-6}$	0.188
37	2130.639	18.793	$< 10^{-6}$	0.191
38	2131.246	18.008	$< 10^{-6}$	0.181

We repeated this calculation for each gameweek for every manager in our dataset and determined both the number of individuals who played the chip that gameweek alongside the average number of points those that did earned from doing so. The corresponding figures are shown in S7, S8, and S9. A fourth chip also exists in the game and is known as the *wildcard*, this chip allows the manager to make as many transfers as they like in the week it is played thus offering a chance to totally redefine their team. The managers receive this chip twice in the season, the first may only be used between gameweeks 1 and 21, while the second in one of the remaining gameweeks. It proves however much more difficult to quantify the return from this chip e.g., one could consider the wildcarded teams return versus their original team in the following m gameweeks, however in practice the manager would make transfers to their original team in the following gameweeks, another issue is the possibility that the manager 'dead-ends' their team up to the week of their wildcard gameweek in the sense that they stop planning for beyond the wildcard opening up the possibility of an extremely biased comparison as the team being changed arguably would not be there without the wildcard. We may still, however, consider the gameweek in which the managers played each of their two chips and this is shown in S11 alongside the quantities themselves in S10. Looking at the point of season in which these chips are used we again notice an evident pattern among the actions of the top managers particularly when the second wildcard chip is considered. It appears as though the strategy of choice for those who finished in the top two tiers was to use their free hit during double gameweek 32, wildcard in gameweek 34, and thus having what they believed to be an optimal squad such that they could optimise their bench boost chip which most played in double gameweek

S6 Table: Summary of players who appeared in the first cluster over the course of the season among the different tiers of managers.

	Tier				
Gameweek	Everyone	10^3	10^4	10^5	10^6
1	Mohamed Salah	Sergio Agüero	Sergio Agüero	Mohamed Salah	Mohamed Salah
2	Mohamed Salah	Sergio Agüero	Sergio Agüero	Sergio Agüero	Mohamed Salah
3	Mohamed Salah	Sergio Agüero	Sergio Agüero	Sergio Agüero	Sergio Agüero
4	Sergio Agüero	Sergio Agüero	Sergio Agüero	Sergio Agüero	Sergio Agüero
5	Sergio Agüero	Sergio Agüero	Sergio Agüero	Sergio Agüero	Sergio Agüero
6	Sergio Agüero	Aaron Wan-Bissaka	Aaron Wan-Bissaka	Sergio Agüero	Sergio Agüero
7	Sergio Agüero	Aaron Wan-Bissaka	Aaron Wan-Bissaka	Sergio Agüero	Sergio Agüero
8	Eden Hazard	Aaron Wan-Bissaka	Aaron Wan-Bissaka	Aaron Wan-Bissaka	Eden Hazard
9	Eden Hazard	Eden Hazard	Eden Hazard	Eden Hazard	Eden Hazard
10	Eden Hazard	Aaron Wan-Bissaka	Aaron Wan-Bissaka	Aaron Wan-Bissaka	Eden Hazard
11	Sergio Agüero	Aaron Wan-Bissaka	Aaron Wan-Bissaka	Sergio Agüero	Sergio Agüero
12	Sergio Agüero	Mohamed Salah	Aaron Wan-Bissaka	Aaron Wan-Bissaka	Sergio Agüero
13	Sergio Agüero	Mohamed Salah	Sergio Agüero	Sergio Agüero	Sergio Agüero
14	Sergio Agüero<br>—<br>—<br>—<br>—	Aaron Wan-Bissaka<br>—<br>—<br>—<br>—	Aaron Wan-Bissaka<br>—<br>—<br>—<br>—	Marcos Alonso<br>Aaron Wan-Bissaka<br>Andrew Robertson<br>Richarlison de Andrade<br>Sergio Agüero	Sergio Agüero<br>—<br>—<br>—<br>—
15	Aaron Wan-Bissaka<br>Callum Wilson<br>Marcos Alonso<br>Richarlison de Andrade	Aaron Wan-Bissaka<br>—<br>—<br>—	Aaron Wan-Bissaka<br>—<br>—<br>—	Aaron Wan-Bissaka<br>—<br>—<br>—	Aaron Wan-Bissaka<br>Callum Wilson<br>Marcos Alonso
16	Aaron Wan-Bissaka<br>Callum Wilson	Aaron Wan-Bissaka<br>—	Aaron Wan-Bissaka<br>—	Aaron Wan-Bissaka<br>—	Callum Wilson<br>—
17	Aaron Wan-Bissaka<br>Marcos Alonso<br>—<br>—	Aaron Wan-Bissaka<br>—<br>—<br>—	Aaron Wan-Bissaka<br>—<br>—<br>—	Aaron Wan-Bissaka<br>—<br>—<br>—	Aaron Wan-Bissaka<br>Callum Wilson<br>Marcos Alonso<br>Pierre-Emerick Aubameyang
18	Aaron Wan-Bissaka<br>—<br>—<br>—	Aaron Wan-Bissaka<br>—<br>—<br>—	Aaron Wan-Bissaka<br>—<br>—<br>—	Aaron Wan-Bissaka<br>—<br>—<br>—	Aaron Wan-Bissaka<br>Andrew Robertson<br>Callum Wilson<br>Marcos Alonso
19	Aaron Wan-Bissaka	Aaron Wan-Bissaka	Aaron Wan-Bissaka	Aaron Wan-Bissaka	Aaron Wan-Bissaka
20	Aaron Wan-Bissaka	Aaron Wan-Bissaka	Aaron Wan-Bissaka	Aaron Wan-Bissaka	Aaron Wan-Bissaka
21	Aaron Wan-Bissaka	Aaron Wan-Bissaka	Aaron Wan-Bissaka	Aaron Wan-Bissaka	Aaron Wan-Bissaka
22	Aaron Wan-Bissaka	Aaron Wan-Bissaka	Aaron Wan-Bissaka	Aaron Wan-Bissaka	Aaron Wan-Bissaka
23	Mohamed Salah	Mohamed Salah	Mohamed Salah	Mohamed Salah	Mohamed Salah
24	Marcus Rashford	Mohamed Salah	Mohamed Salah	Mohamed Salah	Marcus Rashford
25	Mohamed Salah	Mohamed Salah	Mohamed Salah	Aaron Wan-Bissaka	Mohamed Salah
26	Marcus Rashford	Mohamed Salah	Mohamed Salah	Mohamed Salah	Marcus Rashford
27	Paul Pogba	Mohamed Salah	Mohamed Salah	Mohamed Salah	Paul Pogba
28	Raúl Jiménez	Mohamed Salah	Mohamed Salah	Paul Pogba	Raúl Jiménez
29	Paul Pogba	Raúl Jiménez	Paul Pogba	Paul Pogba	Paul Pogba
30	Paul Pogba	Mohamed Salah	Mohamed Salah	Paul Pogba	Paul Pogba
31	Andrew Robertson<br>Eden Hazard<br>Sadio Mané	Mohamed Salah<br>—<br>—	Mohamed Salah<br>—<br>—	Mohamed Salah<br>—<br>—	Mohamed Salah<br>Sadio Mané<br>—
32	Paul Pogba	Sergio Agüero	Sergio Agüero	Sergio Agüero	Paul Pogba
33	Andrew Robertson	Eden Hazard	Eden Hazard	Eden Hazard	Andrew Robertson
34	Raúl Jiménez	Raúl Jiménez	Heung-Min Son	Raúl Jiménez	Raúl Jiménez
35	Raúl Jiménez	Raúl Jiménez	Raúl Jiménez	Raúl Jiménez	Raúl Jiménez
36	Raúl Jiménez	Raúl Jiménez	Raúl Jiménez	Raúl Jiménez	Raúl Jiménez
37	Raúl Jiménez	Raúl Jiménez	Raúl Jiménez	Raúl Jiménez	Raúl Jiménez
38	Sadio Mané	Sergio Agüero	Sergio Agüero	Sadio Mané	Sadio Mané

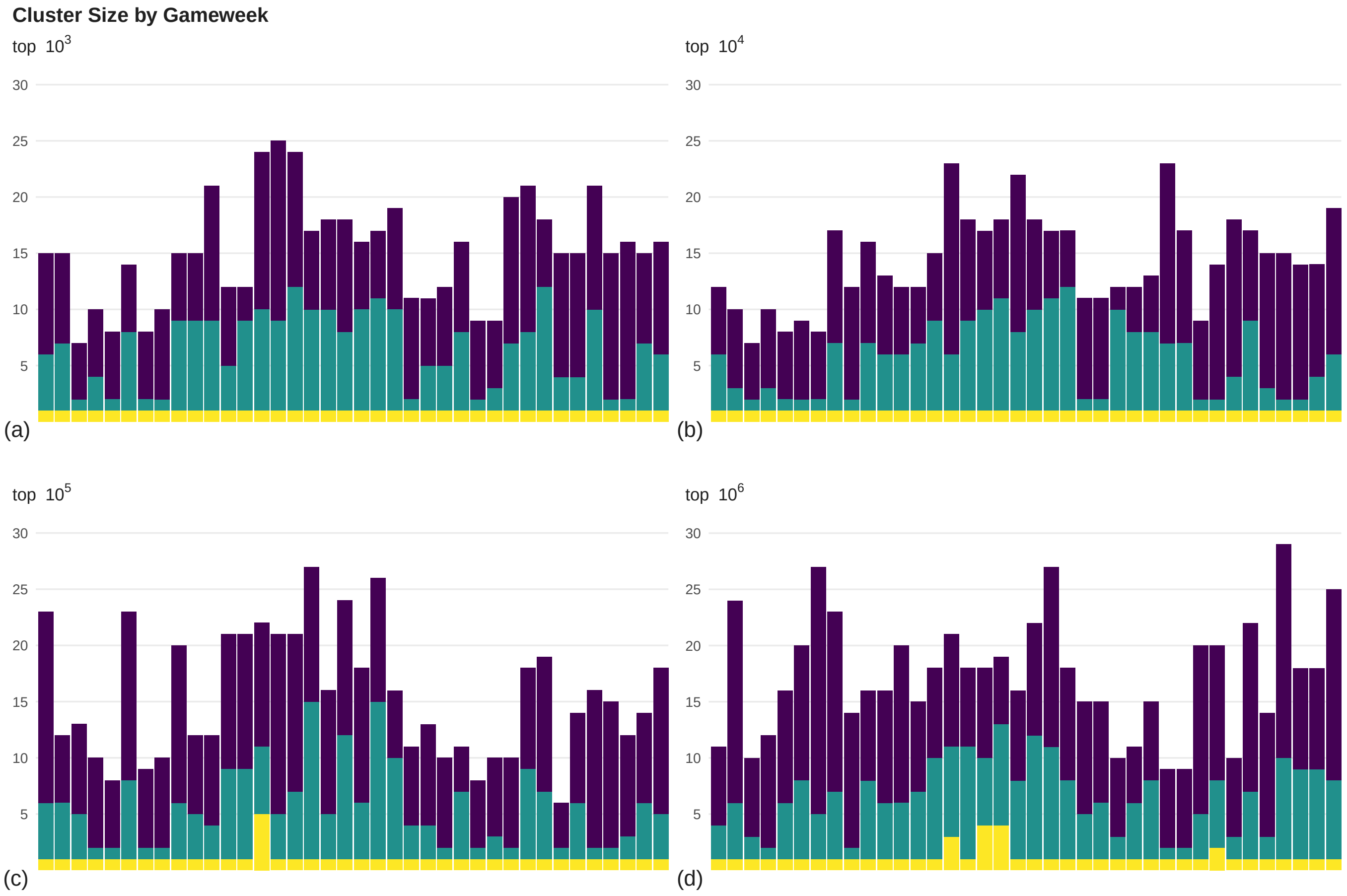

Fig S8. Cluster size analysis. Size of the first three clusters identified by the hierarchical clustering approach described in the main text for each tier. Note all appear to follow a similar pattern.

35.

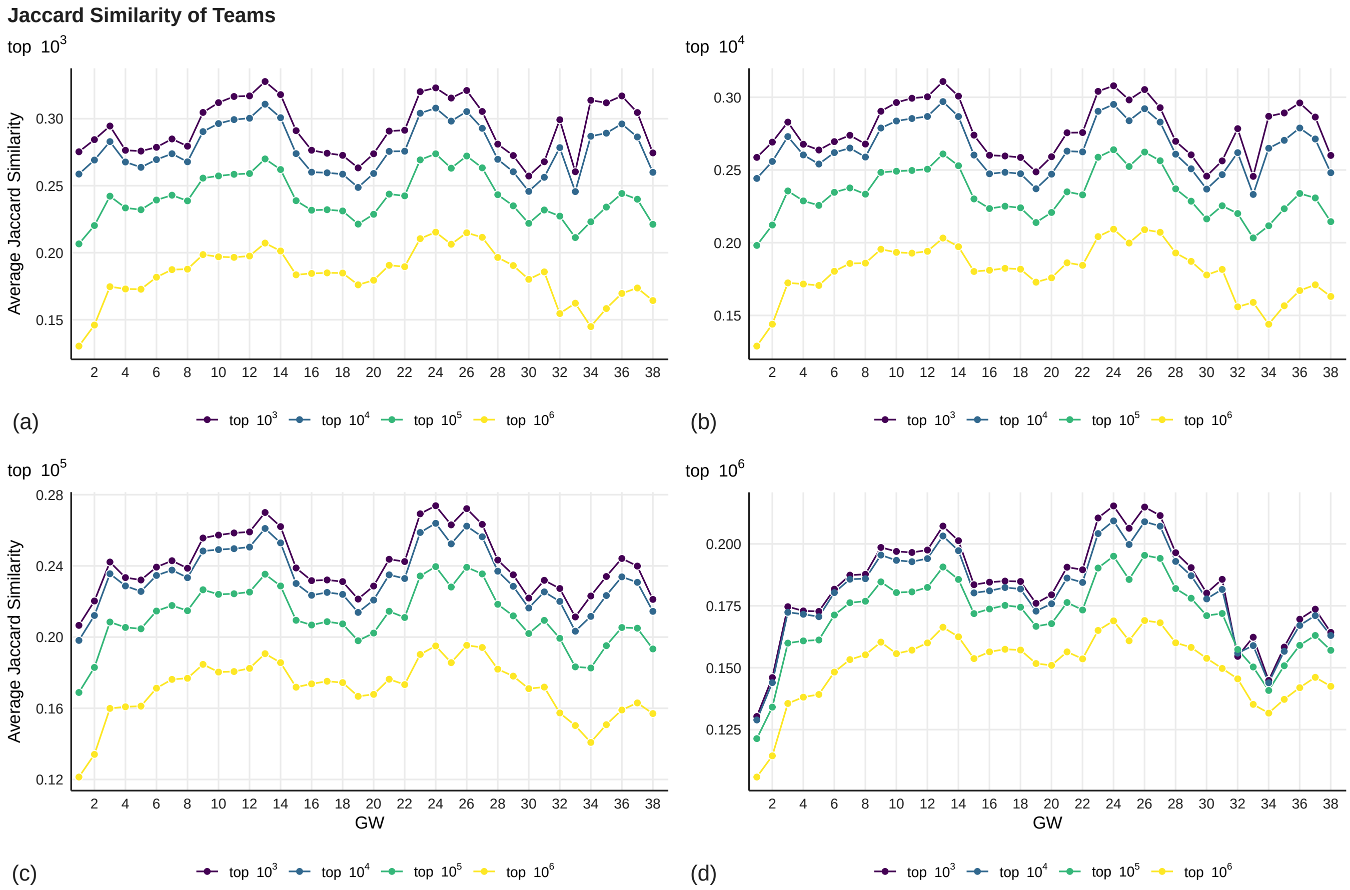

S9 Fig. Jaccard similarity between managers in each tier with those in other tiers. Calculation is repeated as described in Sec. IV C of the main text. The Jaccard similarity of teams in each tier is compared with all other tiers, where we observe a stronger similarity between those in higher tiers indicating that the better managers are more likely to have a similar structure.

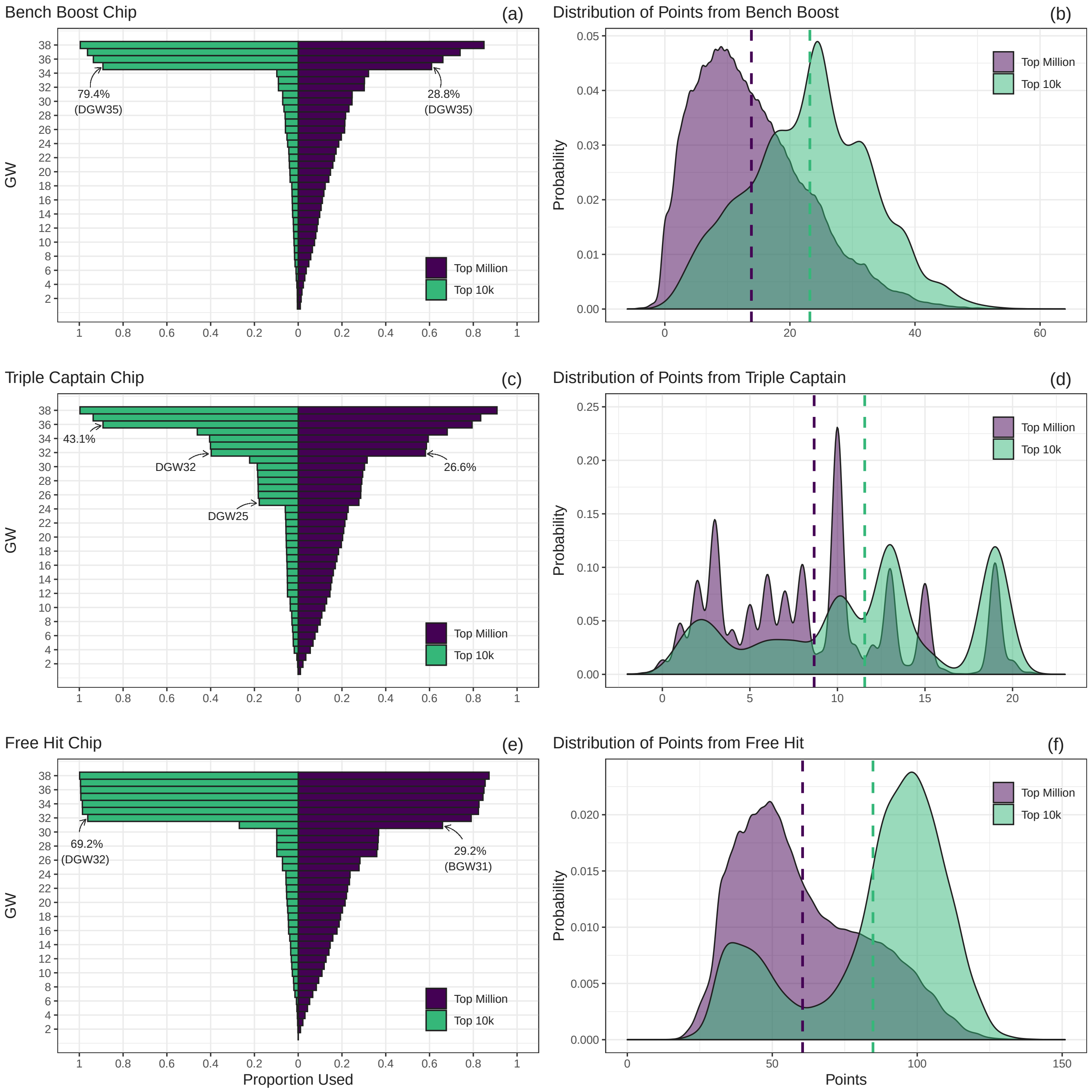

S10 Fig. Summary results for each of the three chips. We show the time of the chips' use along with the points

to the remaining managers (top million) in the dataset. The left panels show the proportion of managers who had used

the large usages in the 'special' gameweeks for each chip. The right panels show the distribution of points received from the chip's use by the two groups of managers, while the mean number of points for each group are also shown by the dashed vertical lines. We comment on the fact that the top 10k received more points on average for each of the three chips.

received by the manager who played them, for manager who finished in the top 10^3 and 10^4 tiers (top 10k) in comparison

the corresponding chip by each GW (the complemen tary cumulative distribution function), particularly highlighting

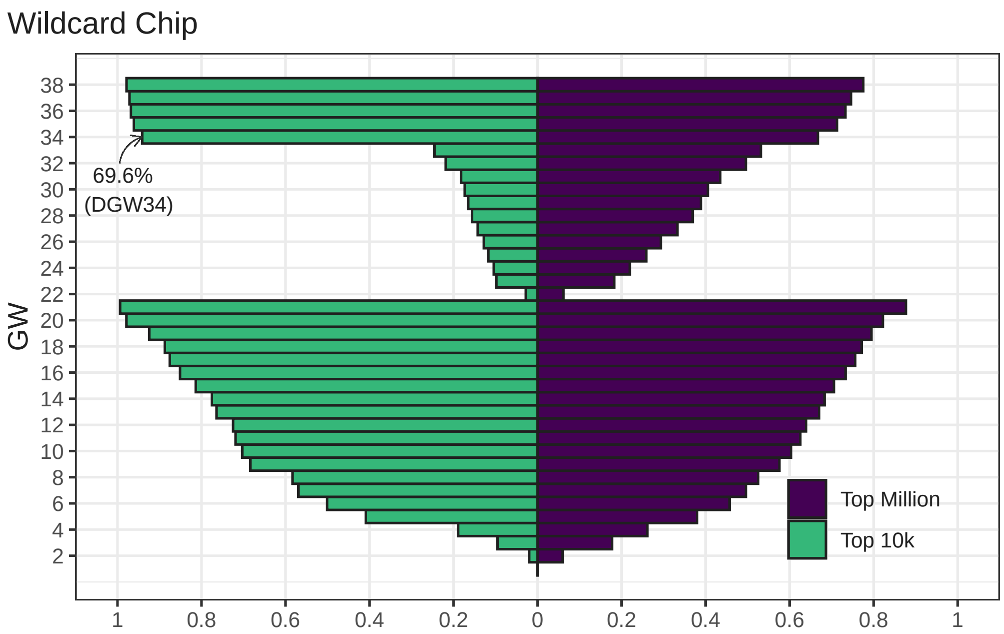

S11 Fig. Analysis of the wildcard chip's use. Fraction of managers in the two groups who had used their wildcard chip by each gameweek. Note that the count resets in gameweek 22 when the chip is replenished.

S7 Table: Usage and Average Points from Bench Boost Chip

	10^3			10^4			10^5			10^6		
GW	Rel. Freq	Cum. Freq	Mean Points	Rel. Freq	Cum. Freq	Mean Points	Rel. Freq	Cum. Freq	Mean Points	Rel. Ferq	Cum. Freq	Mean Points
1	0.003	0.003	20.333	0.004	0.004	21.939	0.006	0.006	16.930	0.010	0.010	11.023
2	0.000	0.003	—	0.000	0.004	4.667	0.001	0.008	7.959	0.004	0.014	7.451
3	0.000	0.003	—	0.000	0.004	8.000	0.001	0.009	9.907	0.004	0.018	10.022
4	0.001	0.004	12.000	0.002	0.006	8.667	0.003	0.012	9.373	0.007	0.025	8.333
5	0.002	0.006	10.000	0.003	0.009	14.042	0.004	0.016	10.143	0.007	0.032	8.783
6	0.000	0.006	—	0.001	0.010	12.500	0.003	0.019	12.431	0.007	0.039	10.543
7	0.003	0.009	13.667	0.005	0.015	12.436	0.008	0.028	11.223	0.012	0.051	10.322
8	0.001	0.010	17.000	0.002	0.017	13.700	0.005	0.033	13.732	0.009	0.060	12.491
9	0.000	0.010	—	0.001	0.018	6.375	0.004	0.037	8.076	0.008	0.068	7.717
10	0.001	0.011	2.000	0.002	0.020	6.214	0.005	0.041	7.409	0.010	0.078	7.376
11	0.001	0.012	7.000	0.001	0.021	9.364	0.002	0.043	9.960	0.006	0.084	9.037
12	0.000	0.012	—	0.001	0.023	11.083	0.003	0.046	10.810	0.007	0.091	10.317
13	0.000	0.012	—	0.001	0.024	12.250	0.002	0.048	9.361	0.005	0.096	8.300
14	0.000	0.012	—	0.003	0.027	7.793	0.005	0.053	9.610	0.007	0.103	8.488
15	0.001	0.013	13.000	0.001	0.028	11.222	0.003	0.056	8.466	0.007	0.110	7.018
16	0.000	0.013	—	0.001	0.029	6.125	0.003	0.059	8.080	0.006	0.116	6.822
17	0.002	0.015	5.500	0.001	0.030	13.000	0.004	0.063	11.663	0.007	0.123	10.209
18	0.000	0.015	—	0.001	0.031	16.444	0.002	0.065	9.754	0.006	0.129	9.492
19	0.005	0.020	16.600	0.008	0.039	15.232	0.012	0.076	11.900	0.017	0.147	10.249
20	0.001	0.021	11.000	0.001	0.040	7.857	0.003	0.079	10.795	0.008	0.155	9.667
21	0.002	0.023	19.000	0.002	0.042	13.333	0.006	0.085	12.410	0.011	0.166	11.187
22	0.000	0.023	—	0.002	0.044	11.538	0.003	0.088	10.874	0.008	0.174	10.083
23	0.001	0.024	13.000	0.001	0.045	6.000	0.004	0.092	9.313	0.008	0.182	8.708
24	0.004	0.028	9.250	0.005	0.051	8.200	0.010	0.102	9.220	0.012	0.194	8.208
25	0.001	0.029	12.000	0.004	0.054	14.467	0.008	0.110	14.635	0.012	0.206	13.299
26	0.009	0.038	11.889	0.007	0.061	8.860	0.013	0.123	7.053	0.015	0.221	6.522
27	0.001	0.039	31.000	0.000	0.061	8.000	0.000	0.123	8.387	0.001	0.223	4.789
28	0.000	0.039	—	0.001	0.062	12.583	0.002	0.125	12.816	0.003	0.226	10.376
29	0.006	0.045	7.167	0.005	0.067	10.326	0.012	0.137	11.184	0.016	0.242	9.979
30	0.004	0.049	19.250	0.006	0.073	13.922	0.010	0.147	12.906	0.014	0.256	11.599
31	0.000	0.049	—	0.000	0.073	8.000	0.000	0.147	5.455	0.001	0.257	3.905
32	0.020	0.069	17.750	0.019	0.093	19.261	0.041	0.188	17.578	0.057	0.314	15.573
33	0.000	0.069	—	0.000	0.093	19.000	0.000	0.189	10.320	0.001	0.315	4.628
34	0.006	0.075	16.333	0.007	0.100	15.082	0.013	0.201	12.463	0.019	0.334	10.647
35	0.856	0.931	27.474	0.787	0.888	25.414	0.594	0.795	23.147	0.257	0.590	18.849
36	0.033	0.964	15.000	0.045	0.933	15.592	0.052	0.847	15.775	0.052	0.642	14.832
37	0.018	0.982	13.778	0.028	0.960	13.233	0.059	0.905	12.486	0.081	0.723	11.447
38	0.017	0.999	11.588	0.035	0.995	10.414	0.074	0.980	9.413	0.113	0.835	8.668

S8 Table: Usage and Average Points from Free Hit Chip

	10^3			10^4			10^5			10^6		
GW	Rel. Freq	Cum. Freq	Mean Points	Rel. Freq	Cum. Freq	Mean Points	Rel. Freq	Cum. Freq	Mean Points	Rel. Freq	Cum. Freq	Mean Points
1	0.000	0.000	—	0.000	0.000	—	0.000	0.000	—	0.000	0.000	—
2	0.001	0.001	67.000	0.001	0.001	84.500	0.003	0.003	78.689	0.011	0.011	71.896
3	0.000	0.001	—	0.002	0.003	53.308	0.004	0.007	55.192	0.012	0.023	53.375
4	0.003	0.004	56.667	0.001	0.004	62.000	0.004	0.011	53.742	0.010	0.033	51.799
5	0.002	0.006	91.000	0.002	0.006	76.706	0.005	0.017	72.022	0.012	0.045	62.225
6	0.001	0.007	64.000	0.002	0.008	64.250	0.004	0.021	60.878	0.010	0.056	56.928
7	0.006	0.013	79.000	0.007	0.015	81.172	0.011	0.031	76.225	0.015	0.070	68.090
8	0.003	0.016	57.667	0.005	0.020	57.667	0.013	0.044	56.857	0.017	0.087	56.142
9	0.000	0.016	—	0.002	0.022	49.214	0.005	0.049	48.288	0.011	0.098	45.351
10	0.006	0.022	97.000	0.005	0.027	88.000	0.010	0.059	83.850	0.015	0.114	78.136
11	0.002	0.024	81.500	0.002	0.030	80.143	0.006	0.066	75.512	0.010	0.124	66.413
12	0.004	0.028	59.250	0.002	0.032	59.062	0.004	0.070	56.708	0.009	0.133	54.179
13	0.001	0.029	90.000	0.003	0.035	69.731	0.008	0.078	66.031	0.014	0.147	61.902
14	0.000	0.029	—	0.000	0.035	61.000	0.002	0.080	58.169	0.006	0.153	57.075
15	0.005	0.034	64.400	0.004	0.039	69.581	0.007	0.087	64.119	0.013	0.166	58.872
16	0.003	0.037	64.000	0.006	0.045	78.957	0.011	0.098	72.813	0.020	0.186	65.906
17	0.000	0.037	—	0.001	0.045	55.714	0.004	0.102	54.592	0.010	0.196	50.406
18	0.000	0.037	—	0.002	0.047	53.231	0.003	0.105	53.367	0.006	0.202	55.985
19	0.001	0.038	94.000	0.002	0.049	84.737	0.005	0.110	80.305	0.010	0.212	74.250
20	0.001	0.039	40.000	0.003	0.052	60.409	0.006	0.117	61.189	0.013	0.225	57.583
21	0.003	0.042	66.333	0.002	0.054	65.429	0.004	0.121	65.541	0.006	0.231	62.016
22	0.000	0.042	—	0.001	0.055	62.750	0.003	0.124	62.424	0.005	0.236	58.843
23	0.002	0.044	94.000	0.002	0.057	75.706	0.005	0.129	76.457	0.009	0.245	69.248
24	0.000	0.044	—	0.000	0.057	47.667	0.001	0.130	52.558	0.003	0.248	47.562
25	0.009	0.053	94.556	0.016	0.074	91.221	0.032	0.162	88.109	0.042	0.290	82.062
26	0.000	0.053	—	0.000	0.074	79.500	0.002	0.163	70.571	0.005	0.295	68.977
27	0.016	0.069	45.000	0.027	0.101	46.240	0.058	0.221	44.662	0.079	0.373	42.860
28	0.000	0.069	—	0.000	0.101	79.500	0.002	0.223	69.053	0.005	0.378	65.063
29	0.000	0.069	—	0.000	0.101	—	0.001	0.223	51.585	0.002	0.380	47.069
30	0.000	0.069	—	0.000	0.101	59.000	0.001	0.224	65.154	0.002	0.382	57.933
31	0.114	0.183	39.561	0.178	0.279	40.827	0.300	0.524	42.048	0.291	0.674	43.879
32	0.788	0.971	99.999	0.681	0.960	98.233	0.399	0.923	96.261	0.103	0.777	92.510
33	0.021	0.992	79.762	0.025	0.985	76.359	0.037	0.961	74.060	0.033	0.809	68.152
34	0.001	0.993	36.000	0.001	0.986	56.400	0.002	0.962	54.674	0.003	0.812	54.866
35	0.003	0.996	84.000	0.007	0.993	77.541	0.013	0.975	76.154	0.019	0.831	74.456
36	0.001	0.997	111.000	0.001	0.993	100.000	0.002	0.977	93.881	0.005	0.835	86.754
37	0.000	0.997	—	0.001	0.994	63.000	0.003	0.980	56.013	0.006	0.841	55.201
38	0.003	1.000	62.000	0.004	0.998	72.833	0.008	0.988	70.057	0.019	0.860	66.632

S9 Table: Usage and Average Points from Triple Captain Chip

	10^3			10^4			10^5			10^6		
GW	Rel. Freq	Cum. Freq	Mean Points	Rel. Freq	Cum. Freq	Mean Points	Rel. Freq	Cum. Freq	Mean Points	Rel. Freq	Cum. Freq	Mean Points
1	0.000	0.000	—	0.001	0.001	8.571	0.003	0.003	7.100	0.011	0.011	5.896
2	0.003	0.003	20.000	0.002	0.002	18.214	0.004	0.007	17.104	0.012	0.023	12.846
3	0.003	0.006	8.000	0.003	0.006	7.000	0.006	0.013	7.015	0.014	0.037	5.971
4	0.006	0.012	6.000	0.013	0.019	6.083	0.017	0.030	6.090	0.021	0.058	5.934
5	0.005	0.017	9.600	0.004	0.023	11.105	0.007	0.037	11.845	0.013	0.071	10.032
6	0.001	0.018	8.000	0.001	0.024	5.000	0.003	0.039	6.167	0.010	0.081	5.868
7	0.001	0.019	8.000	0.003	0.026	8.000	0.004	0.044	8.491	0.012	0.093	8.485
8	0.001	0.020	1.000	0.003	0.030	1.500	0.006	0.049	2.554	0.013	0.106	3.909
9	0.000	0.020	—	0.001	0.031	6.222	0.002	0.052	5.387	0.008	0.114	5.094
10	0.007	0.027	15.000	0.007	0.037	15.000	0.010	0.062	14.529	0.014	0.128	13.513
11	0.001	0.028	13.000	0.000	0.038	15.000	0.003	0.065	12.974	0.009	0.137	11.468
12	0.010	0.038	8.000	0.012	0.050	7.670	0.013	0.078	7.538	0.015	0.152	6.611
13	0.001	0.039	3.000	0.000	0.050	3.000	0.001	0.079	6.391	0.004	0.156	5.252
14	0.000	0.039	—	0.000	0.051	6.500	0.002	0.081	4.089	0.005	0.161	3.565
15	0.000	0.039	—	0.001	0.051	12.000	0.003	0.083	9.582	0.007	0.169	7.424
16	0.000	0.039	—	0.001	0.052	4.500	0.003	0.086	5.891	0.010	0.178	6.045
17	0.001	0.040	5.000	0.001	0.054	4.300	0.003	0.089	6.175	0.008	0.186	5.549
18	0.000	0.040	—	0.001	0.055	4.625	0.002	0.091	6.882	0.008	0.194	7.408
19	0.001	0.041	6.000	0.001	0.056	11.500	0.004	0.096	10.410	0.014	0.208	10.073
20	0.000	0.041	—	0.001	0.057	10.286	0.002	0.098	9.729	0.006	0.214	8.041
21	0.000	0.041	—	0.001	0.058	8.250	0.002	0.100	8.131	0.005	0.219	7.467
22	0.000	0.041	—	0.000	0.058	8.000	0.001	0.101	8.686	0.006	0.225	8.116
23	0.000	0.041	—	0.002	0.060	13.929	0.003	0.104	12.465	0.009	0.234	12.138
24	0.001	0.042	2.000	0.001	0.061	7.182	0.003	0.107	6.650	0.007	0.241	6.418
25	0.152	0.194	14.066	0.115	0.176	12.720	0.077	0.184	12.068	0.046	0.287	12.989
26	0.005	0.199	8.000	0.004	0.180	8.000	0.006	0.190	7.874	0.009	0.296	7.662
27	0.001	0.200	2.000	0.001	0.181	1.400	0.000	0.191	2.310	0.001	0.297	2.208
28	0.000	0.200	—	0.001	0.182	5.571	0.002	0.193	5.558	0.004	0.301	5.509
29	0.001	0.201	3.000	0.001	0.183	2.500	0.002	0.195	2.947	0.004	0.305	2.719
30	0.002	0.203	5.000	0.002	0.185	10.056	0.004	0.199	7.042	0.009	0.314	6.591
31	0.027	0.230	2.000	0.036	0.221	2.581	0.025	0.224	3.083	0.011	0.324	4.741
32	0.121	0.351	9.405	0.182	0.403	9.370	0.291	0.516	9.093	0.264	0.588	8.451
33	0.002	0.353	16.000	0.004	0.406	14.387	0.004	0.520	12.335	0.005	0.593	8.983
34	0.001	0.354	1.000	0.004	0.411	2.216	0.007	0.526	2.938	0.008	0.601	3.580
35	0.056	0.410	6.893	0.056	0.467	6.686	0.077	0.603	6.570	0.088	0.689	6.469
36	0.505	0.915	15.986	0.422	0.889	15.376	0.266	0.869	15.136	0.098	0.787	14.586
37	0.038	0.953	4.368	0.046	0.935	4.205	0.045	0.914	4.106	0.039	0.826	4.165
38	0.045	0.998	6.111	0.062	0.997	6.779	0.074	0.988	6.781	0.075	0.900	6.796

S10 Table: Usage of Wildcard Chip

	10^3		10^4		10^5		10^6	
GW	Rel. Freq	Cum. Freq	Rel. Freq	Cum. Freq	Rel. Freq	Cum. Freq	Rel. Freq	Cum. Freq
1	—	—	—	—	—	—	—	—
2	0.019	0.019	0.020	0.020	0.040	0.040	0.062	0.062
3	0.055	0.074	0.078	0.098	0.104	0.143	0.119	0.181
4	0.093	0.167	0.094	0.192	0.092	0.235	0.083	0.264
5	0.233	0.400	0.218	0.410	0.187	0.422	0.111	0.375
6	0.071	0.471	0.095	0.505	0.091	0.513	0.076	0.451
7	0.083	0.554	0.067	0.571	0.054	0.567	0.037	0.489
8	0.011	0.565	0.014	0.585	0.025	0.593	0.029	0.518
9	0.103	0.668	0.100	0.686	0.075	0.668	0.048	0.566
10	0.014	0.682	0.020	0.705	0.029	0.697	0.028	0.594
11	0.016	0.698	0.016	0.721	0.017	0.714	0.022	0.616
12	0.004	0.702	0.006	0.728	0.010	0.724	0.014	0.630
13	0.031	0.733	0.040	0.768	0.038	0.763	0.030	0.661
14	0.012	0.745	0.011	0.779	0.011	0.774	0.013	0.674
15	0.052	0.797	0.037	0.816	0.032	0.806	0.021	0.695
16	0.045	0.842	0.037	0.852	0.037	0.843	0.027	0.722
17	0.029	0.871	0.024	0.876	0.022	0.865	0.023	0.745
18	0.009	0.880	0.012	0.888	0.014	0.879	0.016	0.760
19	0.037	0.917	0.037	0.925	0.028	0.907	0.023	0.783
20	0.075	0.992	0.052	0.977	0.038	0.945	0.026	0.809
21	0.009	1.001	0.016	0.993	0.034	0.980	0.057	0.866
22	0.016	0.016	0.029	0.029	0.052	0.052	0.063	0.063
23	0.050	0.066	0.072	0.102	0.112	0.164	0.122	0.185
24	0.002	0.068	0.007	0.109	0.019	0.182	0.039	0.223
25	0.005	0.073	0.014	0.122	0.028	0.210	0.040	0.264
26	0.008	0.081	0.011	0.134	0.022	0.233	0.036	0.300
27	0.014	0.095	0.015	0.148	0.026	0.259	0.041	0.341
28	0.008	0.103	0.014	0.162	0.029	0.288	0.037	0.378
29	0.008	0.111	0.009	0.171	0.016	0.303	0.020	0.398
30	0.005	0.116	0.009	0.180	0.013	0.316	0.017	0.415
31	0.003	0.119	0.009	0.189	0.019	0.336	0.030	0.445
32	0.032	0.151	0.037	0.227	0.064	0.399	0.061	0.506
33	0.026	0.177	0.027	0.254	0.041	0.440	0.035	0.541
34	0.793	0.970	0.684	0.938	0.420	0.861	0.106	0.647
35	0.011	0.981	0.021	0.959	0.040	0.900	0.046	0.693
36	0.004	0.985	0.007	0.966	0.014	0.914	0.021	0.714
37	0.002	0.987	0.004	0.970	0.008	0.922	0.014	0.728
38	0.002	0.989	0.008	0.977	0.017	0.939	0.030	0.758
