# Supplementary material for: Identification of skill in an online game: The case of Fantasy Premier League
Source: PLoS One. 2021 Mar 3;16(3):e0246698. doi: 10.1371/journal.pone.0246698 (PMC7928501; doi:10.1371/journal.pone.0246698)
Supplement: Attachment — Submitted filename: Response to Reviewers.pdf [file pone.0246698.s002.pdf]

Dear Prof. Constantinou,

We thank you and the reviewer for the careful reviews and the positive and constructive comments on our manuscript. We hope that the revised manuscript, with changes in blue text, will be considered worthy of publication in the PLOS ONE.

Yours sincerely,

Joseph O'Brien (on behalf of all authors).

Reviewer #1: Very well written paper. I only have minor recommendations, so I recommended publish.

We thank the Reviewer for their extremely positive feedback on our manuscript and recommendation for publication. The reviewer highlights areas in which the clarity of the text could be enhanced: A number of textual edits have been implemented throughout and these changes have resulted in the clarity of the work being considerably improved.

First their statistical performances. It is unclear whether you are talking about players or managers in fantasy.

We appreciate that there may be some confusion due to the managers 'playing' the game which itself is based on the performance of football players. A statement is now included in the introduction which specifically sets out our aim of a statistical analysis of manager performance (rather than players) and we feel this should set the reader on a clearer track for the remainder of the manuscript.

Besides reference 28. A study performed by Easton and Newell in the Journal of Sports Analytics showed that chance doesn't win daily fantasy sports and should be added.

We thank the reviewer for pointing out this reference to us and it has now been included in conjunction with reference 28 to highlight the emerging research into fantasy sports that shows these games involve considerable levels of skill.

Remove the here before introduce in the second paragraph.

Implemented as requested.

The sentence that starts In section IIC3... You may want to add a comment as to why. That is a player plays in more games for the week and thus will earn more points than if the player played in fewer games that week. You should state the claim better than I did though.

We agree with the reviewer that examples of how managers may take advantage of these players appearing multiple times would help the reader and a commentary has been included in the revised manuscript.

With these structures in place....
You comment on hierarchical clustering. I would be more specific. In the details you comment about elbow method and K-means. I would bring these methods into the main body of the paper in all instances. The readers must know the tools you used. Then you can point them to more details in the supplementary sections.

We agree with the reviewer that further commentary on the clustering approaches used in our analysis would help the reader. As such the choice of k-means and the identification of the number of clusters via the elbow method is now explicitly mentioned in Section 3D, and furthermore we now provide more detailed commentary on the approach in the main text rather than in supplemental material (Sec 4D).
